# Homoarginine and inhibition of human arginase activity: kinetic characterization and biological relevance

**DOI:** 10.1038/s41598-018-22099-x

**Published:** 2018-02-27

**Authors:** S. Tommasi, D. J. Elliot, M. Da Boit, S. R. Gray, B. C. Lewis, A. A. Mangoni

**Affiliations:** 1 Department of Clinical Pharmacology, College of Medicine and Public Health, Flinders University and Flinders Medical Centre, Adelaide, Australia; 2 Faculty of Health and Life Sciences, De Montfort University, Leicester, United Kingdom; 3 Institute of Cardiovascular and Medical Sciences, University of Glasgow, Glasgow, United Kingdom; 4 Flinders Centre for Innovation in Cancer, College of Medicine and Public Health, Flinders University, Adelaide, Australia

## Abstract

The inhibition of arginase, resulting in higher arginine (ARG) availability for nitric oxide synthesis, may account for the putative protective effect of homoarginine (HOMOARG) against atherosclerosis and cardiovascular disease. However, uncertainty exists regarding the significance of HOMOARG-induced arginase inhibition *in vivo*. A novel UPLC-MS method, measuring the conversion of ARG to ornithine (ORN), was developed to determine arginase 1 and arginase 2 inhibition by HOMOARG, lysine (LYS), proline (PRO), agmatine (AG), asymmetric dimethylarginine (ADMA), symmetric dimethylarginine (SDMA), and NG-Monomethyl-L-arginine (L-NMMA). Plasma HOMOARG, ARG and ORN concentrations were further measured in 50 healthy older adults >65 years (27 males and 23 females). HOMOARG inhibited arginase 1 with IC_50_ and *K*_i_ values of 8.14 ± 0.52 mM and 6.1 ± 0.50 mM, and arginase 2 with IC_50_ and *K*_i_ values of 2.52 ± 0.01 mM and 1.73 ± 0.10 mM, respectively. Both arginase isoforms retained 90% activity vs. control when physiological HOMOARG concentrations (1–10 µM) were used. In partial correlation analysis, plasma HOMOARG was not associated with ARG (P = 0.38) or ARG/ORN ratio (P = 0.73) in older adults. Our results suggest that arginase inhibition is unlikely to play a significant role in the reported cardio-protective effects of HOMOARG.

## Introduction

Recent human studies have reported an inverse correlation between the serum and plasma concentrations of homoarginine (HOMOARG), a basic amino acid and analogue of arginine (ARG), and cardiovascular risk. Chemically, HOMOARG differs from ARG by a single methylene-group extension of the side-chain. Clinically, low serum concentrations of HOMOARG are associated with renal dysfunction, increased cardiovascular risk and mortality^1–7^. High serum HOMOARG concentrations have also been associated with enhanced endothelial function in the mother during pregnancy^8^. It has been speculated that HOMOARG exerts protective effects in the cardiovascular system *via* inhibition of the enzyme arginase. Arginase is an enzyme of the urea cycle that catalyses the conversion of ARG to ornithine (ORN) and urea, thus playing a key role in nitrogen metabolism. There are two arginase isoforms in mammals, arginase 1 and arginase 2, with different tissue and cellular distributions. The role of arginase 1 is particularly important in liver and blood cells, while arginase 2 is a key enzyme in the kidney^9^. HOMOARG-mediated arginase inhibition would lead to the accumulation of the substrate ARG, and a consequent increase in nitric oxide (NO) synthesis by the NO synthase enzymes^1,7^. The latter would provide salutary effects in terms of vascular homeostasis and atheroprotection.

Several studies have previously investigated arginase 1 and arginase 2 enzyme kinetics, identified alternative substrates, and characterised the inhibitory potential of endogenous compounds^10–19^. However, the results of studies investigating the effect of HOMOARG on arginase activity have been contradictory. Both Reczkowski and Ash^11^ and Hunter and Downs^14^ reported that HOMOARG is an alternative substrate for arginase 1. However, other studies have shown that HOMOARG is an arginase 1 inhibitor, without substrate activity^10,12,20^. Similarly, HOMOARG was found to be an inhibitor, but not a substrate, of arginase 2^17^. More recently, Michel has reproposed HOMOARG as a potential arginase substrate^19^.

In view of the conflicting results of previous studies, clarification of the role of HOMOARG in modulating the activity of the arginase isoforms 1 and 2 will have significant biological and clinical relevance. We sought to address this issue by studying the effects of HOMOARG, in addition to other endogenous molecules such as lysine (LYS), proline (PRO), agmatine (AG), asymmetric dimethylarginine (ADMA), symmetric dimethylarginine (SDMA), and NG-Monomethyl-L-arginine (L-NMMA), on arginase 1 and 2 activity. LYS was selected as a positive control for arginase inhibition, as it is the prototypic arginase inhibitor^10–15^. In contrast, ADMA, SDMA, L-NMMA, important modulators of the NO pathway together with ARG and HOMOARG, have failed to show inhibitory potential when incubated with arginase, and were selected as negative controls^18,19^. Additionally, PRO and AG, previously described as poor arginase inhibitors^10,11,14–17,21^, were included in this study. The concentration range used for the compounds tested was chosen in order to cover their physiological concentrations found in plasma and up to 10,000-fold their circulating concentrations.

Our *in vitro* experimentation utilised a highly sensitive and specific UPLC-MS method to measure arginase activity, from an expression system that better represents physiological conditions. Specifically, we incubated our samples in phosphate buffer, abundant in living organism, instead of the commonly reported tris-buffer, and we used un-purified cell lysate as the source of protein. Lysates were obtained from cells overexpressing arginase 1 or arginase 2 in the absence of EDTA to maintain the cellular concentrations of divalent ions, removing the need for manganese supplementation. We also investigated the inhibitory potential of HOMOARG on arginase activity *in vivo* by assessing the associations between HOMOARG concentrations and the ARG/ORN ratio in a cohort of healthy older adults.

## Results

### Cloning and expression of arginase 1 and arginase 2

**Figure 1:**
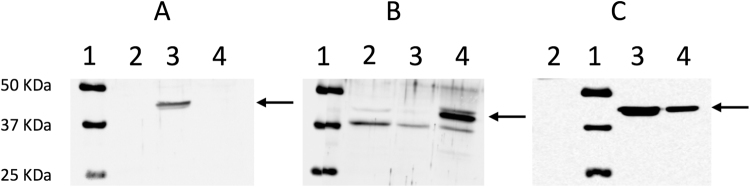
Expression of recombinant arginase 1 and arginase 2 in HEK293T cell lysate as shown by representative western blots performed using **(A)** anti-arginase 1, **(B)** anti-arginase 2, and **(C)** anti-FLAG primary antibodies, respectively. Each blot shows: molecular markers (lane 1), untransfected HEK293T cell lysate (lane 2, 100 µg), arginase 1 lysate (lane 3, 30 µg), and arginase 2 lysate (lane 4, 30 µg).

HEK293T cells stably expressing recombinant human arginase 1 or arginase 2 were analysed using immunological detection. Both arginase enzymes were observed at an apparent molecular weight of 40 kDa (Fig. 1 and Supplementary File) as expected according to the antibodies manufacturer. Immunoreactive bands were not observed when probing arginase 1 lysates with anti-arginase 2 antibody, and vice versa.

### UPLC-MS analysis of ORN

**Figure 2:**
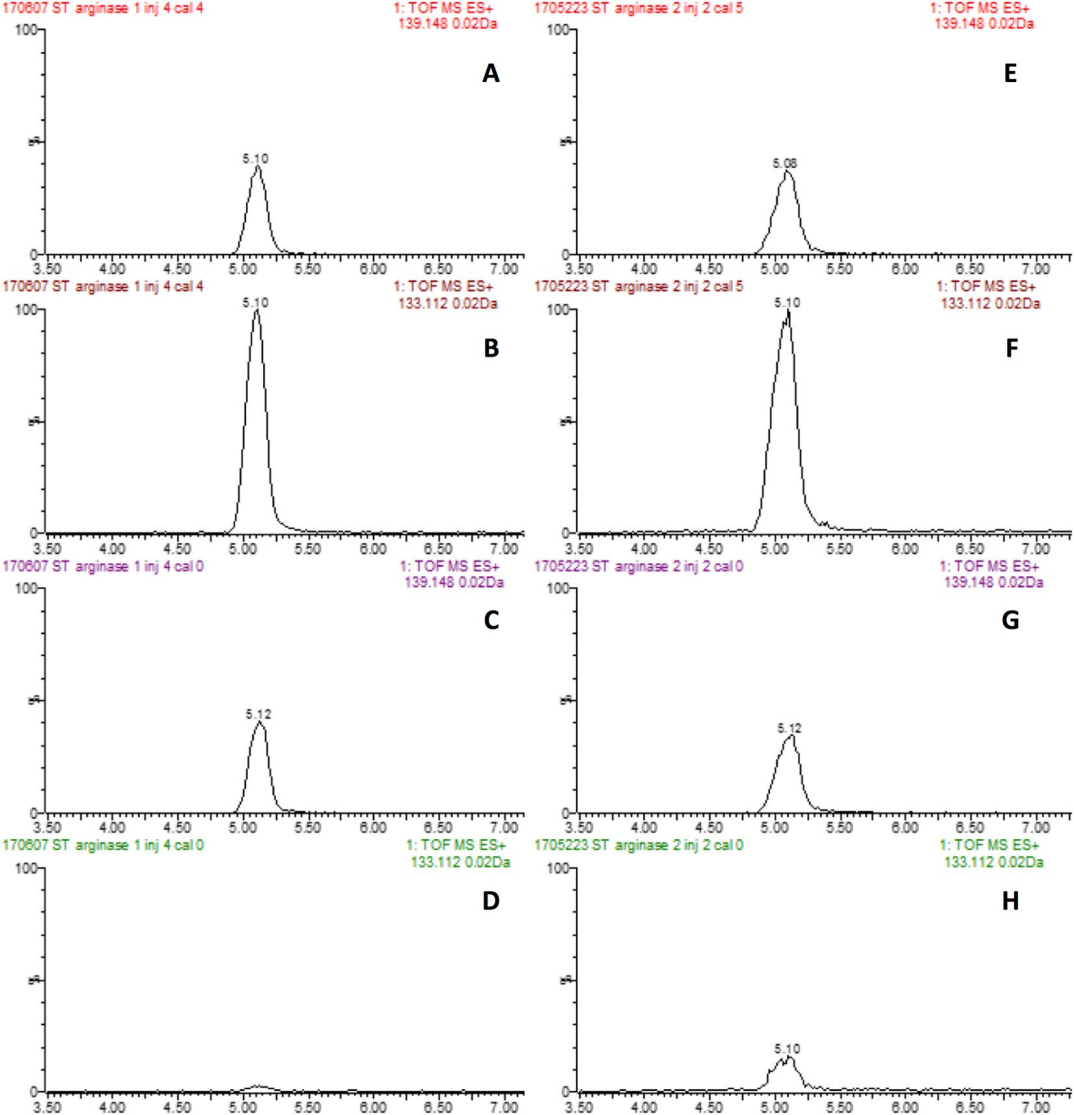
Representative chromatograms for ORN (**B**,**D**,**F** and **H**) and internal standard ORN–d6 (**A**,**C**,**E** and **G**) extracted at 133.112 and 139.148 Da corresponding to molecular ion of ORN and ORN–d6 respectively. Chromatograms are shown for ORN in arginase 1 calibrator 0 (**C** and **D**) and calibrator 4 (**A** and **B**), comprising 0.05 mg/mL arginase 1 HEK293T lysate, 0.05 M phosphate buffer, 3 mM ARG, 10 µL of 1200 µM ORN–d6 and 0 µM ORN (**D**) or 200 µM ORN (**B**). The corresponding internal standard chromatograms are shown in panels **C** and **A**. Similarly, chromatograms are shown for ORN in arginase 2 calibrator 0 (**G** and **H**) and calibrator 5 (**E** and **F**), comprising 0.1 mg/mL arginase 2 HEK293T lysate, 0.05 M phosphate buffer, 2 mM ARG, 10 µL of 300 µM ORN–d6 and 0 µM ORN (**H**) or 50 µM ORN (**F**). The corresponding internal standard chromatograms are shown in panels **G** and **E**. The small peak observed for ORN in calibrator 0 (**D** and **H**) arises from very low concentrations of endogenous ORN present in the HEK293T lysate.

Extracted ion chromatograms (EICs) were obtained with a mass window of 0.02 Da from total ion chromatogram (TIC) using m/z of 133.11 and 139.15 corresponding to the parent ions ([M + H]^+^) of ORN and ORN-d6 respectively (Fig. 2). Calibration curves were obtained by plotting the peak area ratio ORN to internal standard versus the standard concentration.

**Figure 3:**
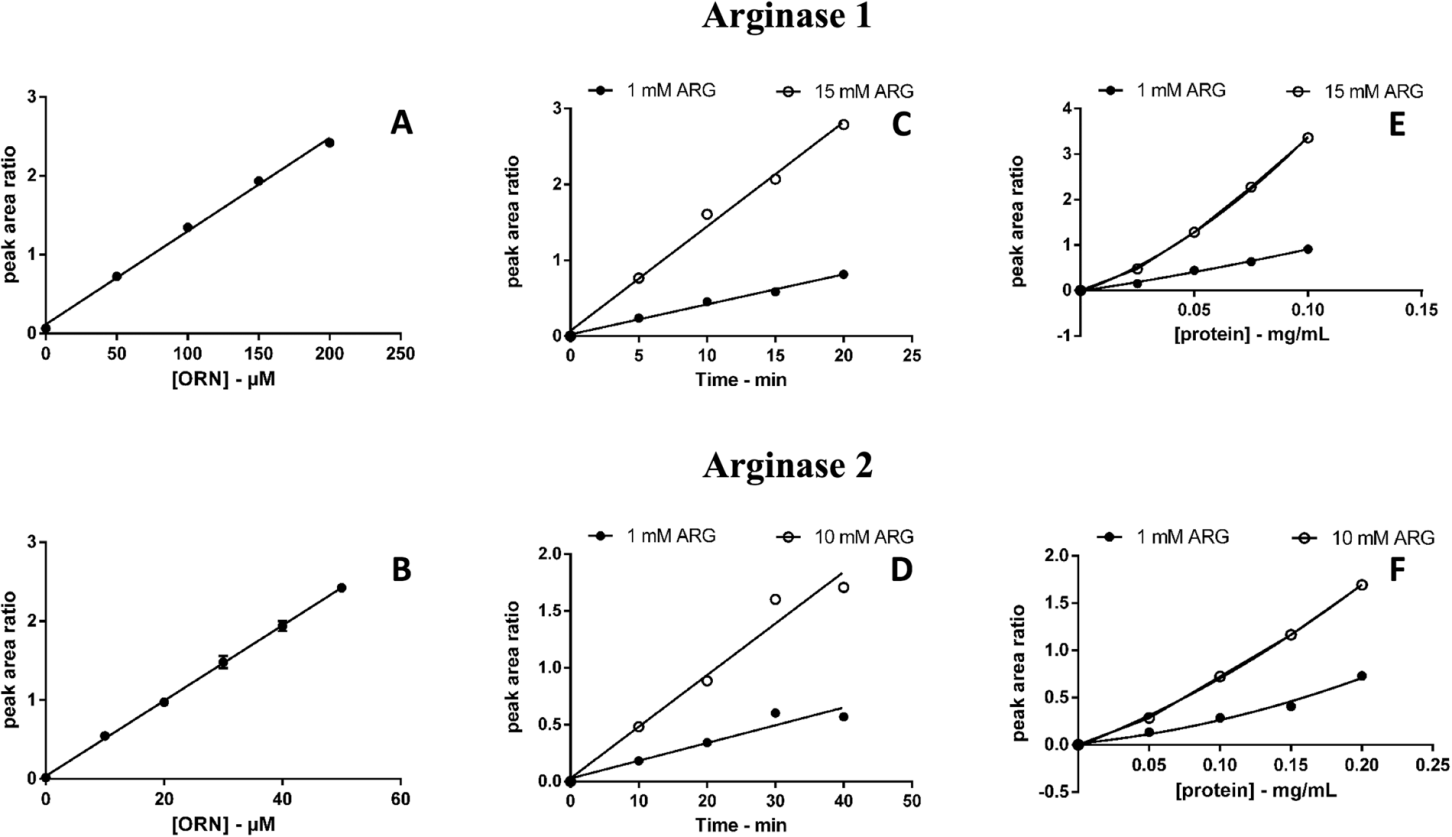
Calibration curves, time and protein plots for arginase 1 (**A**,**C** and **E**) and arginase 2 (**B**,**D**,**F**). ORN calibration curves (**A** and **B**) were constructed by plotting the peak area ratio ORN to ORN-d6 versus the ORN concentration. Each data point represents the mean of three injections. Error bars represent the standard error of the mean. Time (**C** and **D**) and protein (**E** and **F**) data were collected in singlicate.

ORN quantification was linear between 0 and 200 µM (Fig. 3), with a limit of detection of 3 and 0.1 µM for arginase 1 and arginase 2 assays, respectively, and a limit of quantitation of 10 and 0.3 µM for arginase 1 and arginase 2, respectively. The limit of detection was calculated as the ORN concentration corresponding to 3:1 signal to noise ratio, whereas the limit of quantitation was calculated as the ORN concentration corresponding to 10:1 signal to noise ratio.

### Characterisation of arginase 1 and arginase 2 kinetic parameters

Linear conditions for the conversion of ARG into ORN were observed up to 20-min time for arginase 1 and up to 40-min time for arginase 2 (Fig. 3). ORN conversion was not linear across the range of protein concentrations investigated, with the same trend observed for both arginase 1 and arginase 2 (Fig. 3). Reproducibility of ORN formation was assessed for 6 replicates with substrate concentrations of 1 and 10 mM for both arginase 1 and arginase 2 (Table 1).

**Table 1:** Reproducibility of ORN formation.

Isoenzyme	ARG/mM	ORN/µM	% CV
Arginase 1	1	56.4	6.3
Arginase 1	10	153.5	2.9
Arginase 2	1	11.7	4.1
Arginase 2	10	21.1	4.2

As the rate of arginase 1-mediated ORN formation from ARG was 20% higher in absence of manganese supplementation, manganese supplementation was not used in our experiments. Omission of manganese did not influence the shape of the rate-concentration curve (data not shown) and hence it was not expected to influence the kinetic parameters K_m_ and K_i_.

The kinetic behaviour of the arginase 1 expression system was characterised at 0.05 mg/mL protein and 10-min incubation time. The kinetic parameters were derived from non-linear least squares fitting of experimental data for ARG conversion to ORN. For arginase 1, mean *K*_m_ and V_max_ values were 3.3 ± 0.2 mM and 34 ± 1 nmol·min^−1^·mg^−1^, respectively. Characterisation of ARG conversion to ORN by arginase 2 was performed at 0.1 mg/mL protein with a 20-min incubation time. Mean *K*_m_ and V_max_ values were 1.9 ± 0.1 mM and 883 ± 16 pmol·min^−1·^mg^−1^, respectively.

**Figure 4:**
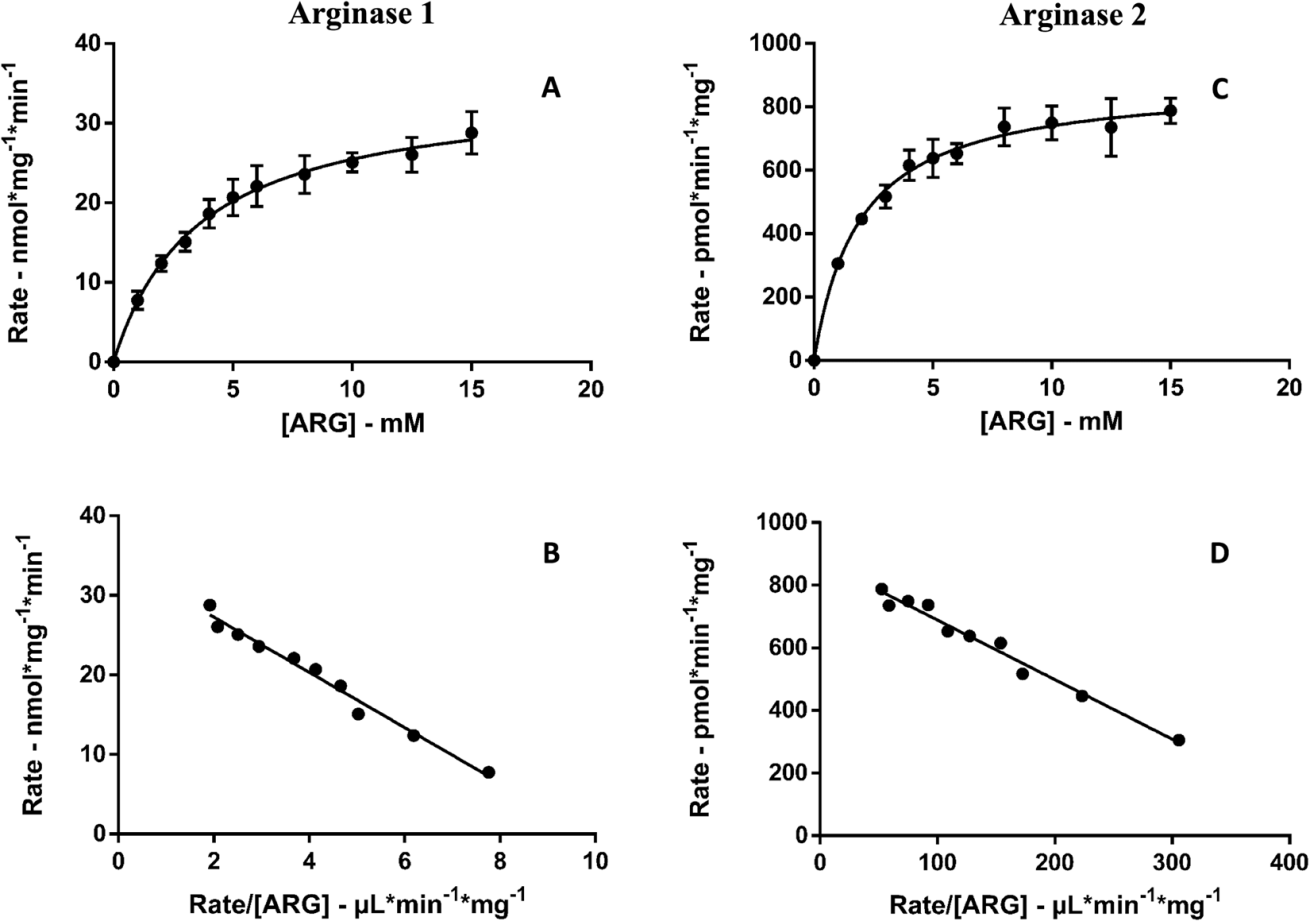
Kinetic plots representing the conversion of ARG to ORN by arginase 1 (**A** and **B**) and by arginase 2 (**C** and **D**). Each data point is the mean of three singlicate experiments and error bars represent the standard error. The data is represented as an Eadie-Hoffstee transform in **B** and **D**. The Michaelis-Menten fit is shown as a solid line in panel **A** and **C**.

Derived *K*_m_ and V_max_ values are reported in Table 2 and Fig. 4.

**Table 2:** Derived kinetic parameters for the conversion of ARG to ORN by arginase 1 and arginase 2.

Enzyme	Parameter	Expt 1	Expt 2	Expt 3	Mean
Arginase 1	K_m_ (mM) ± SE	3.4 ± 0.4	3.8 ± 0.2	3.5 ± 0.2	3.3 ± 0.2
	95% confidence interval K_m_	2.6–4.2	3.4–4.2	3.0–4.0	2.9–3.8
	V_max_ (nmol·min^−1^·mg^−1^) ± SE	37 ± 1	32 ± 1	35 ± 1	34 ± 1
	95% confidence interval V_max_	34–40	31–33	33–36	32–35
	F statistic	1360	3194	3419	4346
	R-squared	0.9927	0.9969	0.9971	0.9977
	SE of fit	0.8875	0.4901	0.5173	0.4581
Arginase 2	K_m_ (mM) ± SE	2.4 ± 0.1	1.7 ± 0.1	1.8 ± 0.1	1.9 ± 0.1
	95% confidence interval K_m_	2.2–2.5	1.5–1.9	1.7–1.9	1.6–2.2
	V_max_ (pmol·min^−1^·mg^−1^) ± SE	984 ± 8	837 ± 12	834 ± 5	883 ± 16
	95% confidence interval V_max_	967–1002	810–864	822–846	848–918
	F statistic	1833	3390	379	2662
	R-squared	0.9946	0.9971	0.9743	0.9963
	SE of fit	20.5065	13.5723	38.162	15.5307

Kinetic constants (*K*_*m*_, V_max_) for ORN formation were derived from fitting the Michaelis-Menten equation to experimental data using the nonlinear curve fitting software EnzFitter.

SE: standard error.

### Arginase 1 and arginase 2 inhibition

#### Concentration-dependent effects

Data represent the mean of two singlicate experiments. A concentration-dependent inhibition of arginase 1 by LYS and HOMOARG was observed at both substrate concentrations of 3 mM (*K*_m_) and 100 µM (physiological concentration of ARG in plasma). LYS, at concentrations of 1 and 10 mM, showed 39 and 78% inhibition of arginase 1 activity at *K*_m_ concentration of the substrate, and 44 and 81% inhibition at physiological ARG concentrations. When the experiments were performed at *K*_m_, HOMOARG, at a concentration of 1 mM, inhibited arginase 1 activity by 14%, whereas a concentration of 10 mM resulted in 50% inhibition of the enzyme. At 100 µM ARG concentration, arginase 1 inhibition by 1 and 10 mM HOMOARG was 30 and 76%, respectively. Notably, no significant inhibitory effects of arginase 1 activity were observed with physiological concentrations of LYS, 100–1000 µM^22^, and HOMOARG, 1–10 µM^23^, respectively. Conversely, at the concentrations tested, PRO, AG, ADMA, L-NMMA and SDMA were poor arginase 1 inhibitors at both ARG concentrations (Table 3). An analogous trend emerged for arginase 2 inhibition by LYS and HOMOARG, while PRO, AG and methylated arginines were confirmed as poor inhibitors of each arginase isoenzymes. Experiments performed using arginase 2 and 2 mM ARG resulted in 18 and 94% inhibition by HOMOARG at the concentration of 1 and 10 mM, and 42 and 67% inhibition by LYS at 1 and 10 mM. At 100 µM ARG concentration, the conversion of ARG to ORN by arginase 2 was inhibited by 47 and 88% in the presence of 1 and 10 mM HOMOARG, and there was 44 and 88% inhibition by 1 and 10 mM LYS (Table 3). Similarly to arginase 1, no significant inhibition of arginase 2 was observed at physiological LYS and HOMOARG concentrations.

**Table 3:** Concentration-dependent inhibition of the conversion of ARG to ORN by arginase 1 and arginase 2. Each data point is the mean of two independent experiments.

Isoenzyme	ARG	Inhibitor	% of control activity at the corresponding [Inhibitor] ± SE					
			0.1 µM	1 µM	10 µM	100 µM	1,000 µM	10,000 µM
Arginase 1	3 mM	HOMOARG	—	106 ± 7	110 ± 1	96 ± 6	86 ± 13	50 ± 1
		LYS	—	108 ± 13	103 ± 10	82 ± 4	63 ± 8	22 ± 1
		PRO	—	100 ± 1	113 ± 26	106 ± 19	100 ± 17	79 ± 20
		AG	—	84 ± 13	100 ± 3	100 ± 8	98 ± 13	78 ± 7
		ADMA	98 ± 7	89 ± 2	90 ± 1	86 ± 4	69 ± 4	—
		SDMA	113 ± 16	93 ± 5	92 ± 2	84 ± 1	73 ± 5	—
		L-NMMA	106 ± 15	107 ± 11	95 ± 9	94 ± 8	97 ± 11	—
	100 µM	HOMOARG	—	112 ± 4	107 ± 4	85 ± 1	70 ± 1	24 ± 8
		LYS	—	88 ± 21	89 ± 10	105 ± 18	56 ± 5	19 ± 1
		PRO	—	115 ± 1	110 ± 1	110 ± 6	104 ± 3	54 ± 7
		AG	—	94 ± 7	82 ± 5	110 ± 24	84 ± 8	72 ± 3
		ADMA	102 ± 9	109 ± 4	120 ± 12	109 ± 8	87 ± 1	—
		SDMA	96 ± 3	113 ± 22	104 ± 4	106 ± 17	69 ± 10	—
		L-NMMA	95 ± 2	91 ± 6	104 ± 11	95 ± 6	116 ± 1	—
Arginase 2	2 mM	HOMOARG	—	95 ± 3	106 ± 2	94 ± 2	72 ± 1	15 ± 8
		LYS	—	88 ± 6	82 ± 6	84 ± 5	58 ± 1	33 ± 4
		PRO	—	98 ± 1	97 ± 7	96 ± 12	98 ± 10	75 ± 8
		AG	—	104 ± 4	96 ± 3	103 ± 3	96 ± 3	87 ± 8
		ADMA	80 ± 7	84 ± 12	92 ± 12	90 ± 14	87 ± 15	—
		SDMA	96 ± 2	100 ± 3	107 ± 1	102 ± 1	96 ± 1	—
		L-NMMA	96 ± 7	105 ± 4	109 ± 8	104 ± 3	118 ± 4	—
	100 µM	HOMOARG	—	107 ± 2	105 ± 5	100 ± 5	53 ± 4	12 ± 5
		LYS	—	92 ± 6	97 ± 5	78 ± 11	56 ± 14	12 ± 4
		PRO	—	92 ± 10	99 ± 1	99 ± 2	92 ± 7	77 ± 16
		AG	—	103 ± 4	98 ± 7	92 ± 6	78 ± 5	58 ± 1
		ADMA	115 ± 7	112 ± 13	109 ± 9	94 ± 14	104 ± 3	—
		SDMA	90 ± 2	96 ± 1	95 ± 3	90 ± 1	89 ± 1	—
		L-NMMA	101 ± 1	103 ± 2	106 ± 1	106 ± 9	111 ± 4	—

The inhibitory potential was fully characterised for compounds showing 50% inhibition or more at the highest concentration used in these preliminary experiments, thus further characterisation was undertaken for LYS and HOMOARG only.

#### IC_50_ and K_i_ values determination for LYS and HOMOARG

**Figure 5:**
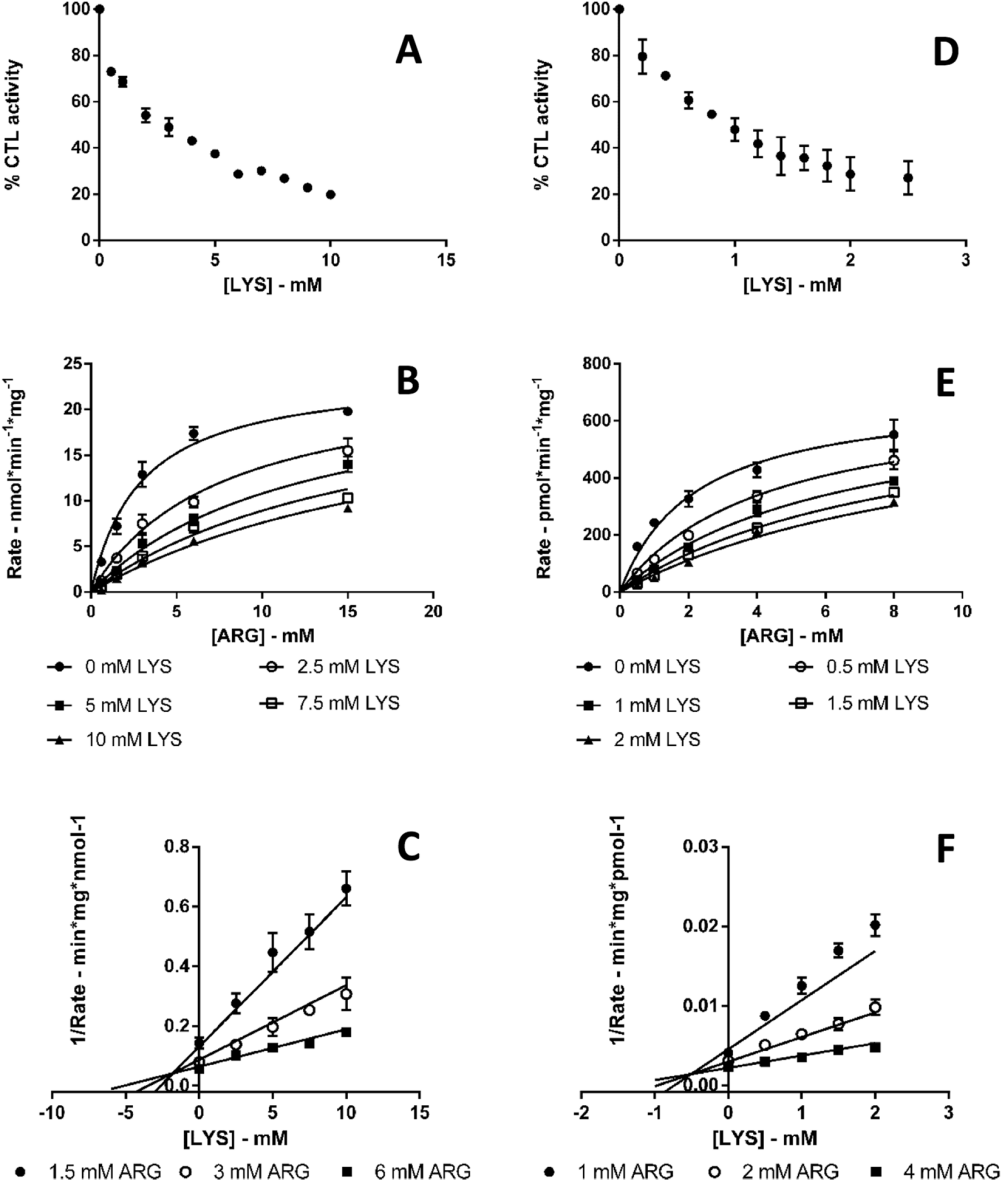
Kinetic plots for arginase 1 and arginase 2 inhibition by LYS. Error bars represent the standard error of the mean. (**A**) Arginase 1 inhibition by LYS IC_50_ plot. Each data point is the mean of two singlicate experiments, (**B**) Arginase 1 inhibition by LYS K_i_ plot. Each data point is the mean of three singlicate experiments and the competitive model fit is represented as solid lines, (**C**) Representative Dixon plot of the inhibition of arginase 1 by LYS. Each data point is the mean of three singlicate experiments and the competitive model fit is shown as solid lines, (**D**) Arginase 2 inhibition by LYS IC_50_ plot. Each data point is the mean of two singlicate experiments, (**E**) Arginase 2 inhibition by LYS K_i_ plot. Each data point is the mean of three singlicate experiments and the competitive model fit is shown as solid lines, (**F**) Representative Dixon plot of the inhibition of arginase 2 by LYS. Each data point is the mean of three singlicate experiments and the competitive model fit is represented as solid lines.

**Figure 6:**
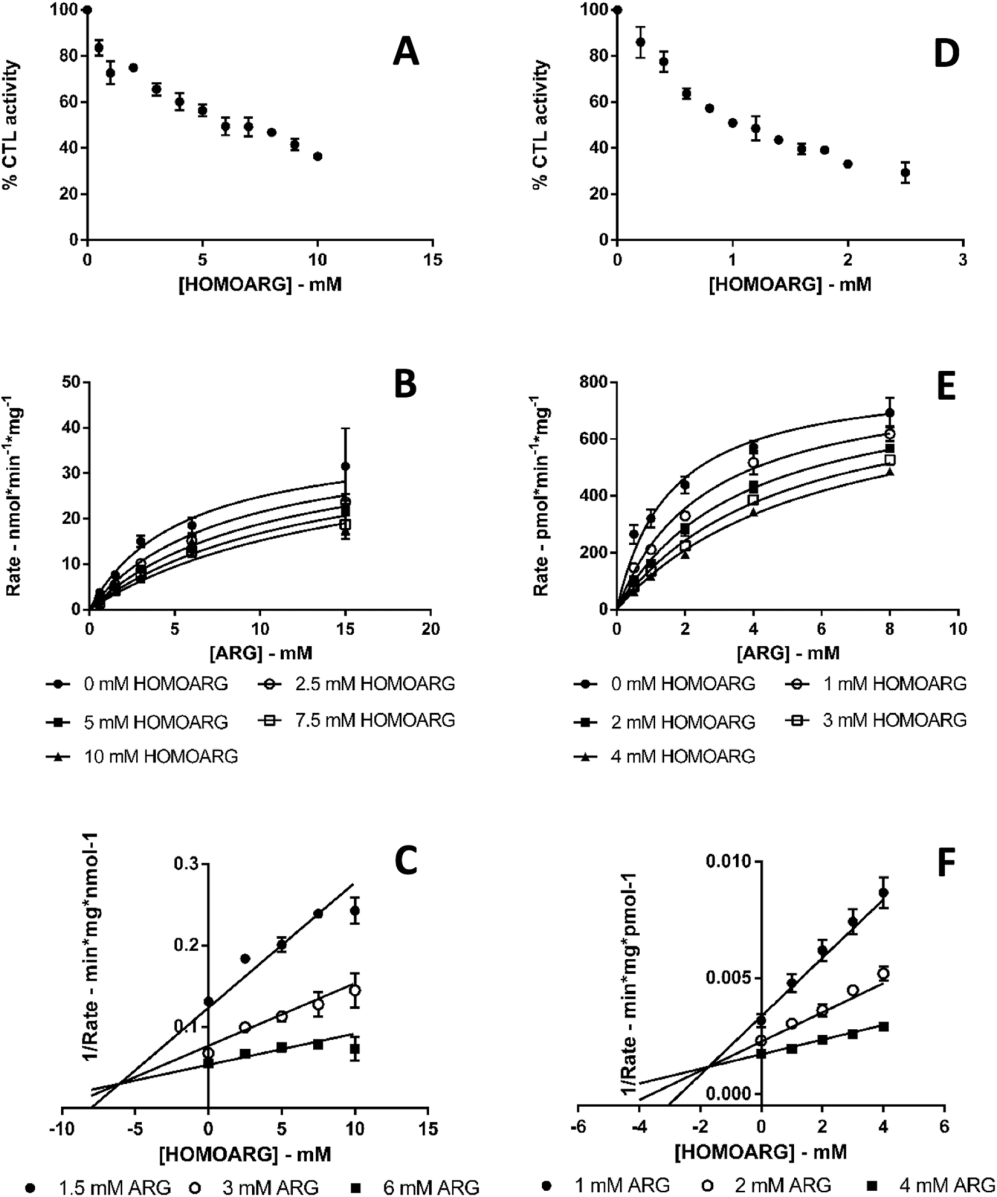
Kinetic plots for arginase 1 and arginase 2 inhibition by HOMOARG. Error bars represent the standard error of the mean. (**A**) Arginase 1 inhibition by HOMOARG IC_50_ plot. Each data point is the mean of two singlicate experiments, (**B**) Arginase 1 inhibition by HOMOARG K_i_ plot. Each data point is the mean of three singlicate experiments and the competitive model fit is represented as solid lines, (**C**) Representative Dixon plot of the inhibition of arginase 1 by HOMOARG. Each data point is the mean of three singlicate experiments and the competitive model fit is shown as solid lines, (**D**) Arginase 2 inhibition by HOMOARG IC_50_ plot. Each data point is the mean of two singlicate experiments, (**E**) Arginase 2 inhibition by HOMOARG K_i_ plot. Each data point is the mean of three singlicate experiments and the competitive model fit is shown as solid lines, (**F**) Representative Dixon plot of the inhibition of arginase 2 by HOMOARG. Each data point is the mean of three singlicate experiments and the competitive model fit is represented as solid lines.

A full characterisation of the kinetic profile of arginase 1 and arginase 2 inhibition by LYS and HOMOARG was performed. The derived IC_50_ and kinetic constants are reported in Table 4. Experimental data for the inhibition of arginase 1 and 2 by LYS and HOMOARG were poorly fit by the equations for uncompetitive, noncompetitive and mixed inhibition. By contrast, data fitted well with the equation for the competitive model. Fitted values and statistical descriptors are given in Table 4 and fitted models, plotted with experimental data, are shown in Figs 5 and 6.

**Table 4:** Derived parameters for arginase 1 and arginase 2 inhibition by LYS and HOMOARG

Enzyme	Inhibitor	IC_50_ ( ± SE)/mM	F-value	R^2^	*K*_i_ ( ± SE)/mM	F-value	R^2^
Arginase 1	LYS	3.64 ± 0.20	1491	0.993	1.79 ± 0.01	1358	0.992
	HOMOARG	8.14 ± 0.52	584	0.983	6.10 ± 0.50	1997	0.995
Arginase 2	LYS	0.88 ± 0.01	2028	0.995	0.50 ± 0.03	1268	0.991
	HOMOARG	2.52 ± 0.01	1691	0.994	1.73 ± 0.10	4114	0.997

SE: standard error.

### HOMOARG as a substrate for arginase 1 or arginase 2

The formation of LYS as a potential product of the reaction of HOMOARG with arginase 1 and arginase 2 was determined by UPLC-MS. Extracted ion chromatograms (EICs) were obtained with a mass window of 0.02 Da from total ion chromatograms (TIC) using m/z of 147.13 corresponding to the parent ion ([M + H] ^+^) for LYS. The retention time for LYS was 5.98 minutes as confirmed using pure LYS. LYS quantification was linear between 0 and 100 µM, with a limit of detection of 0.3 µM and a limit of quantitation of 1 µM (determined using a signal to noise ratio of 3:1 and 10:1, respectively) for the arginase 1 and arginase 2 assays. We could not detect the formation of LYS from HOMOARG in our experiments (data not shown).

### Plasma homoarginine concentrations and arginine/ornithine ratio in humans

The median age of the study population was 70 years (IQR 67–73). Mean baseline plasma concentrations of HOMOARG, ARG, and ORN were 2.36 ± 0.76 µmol/L, 305 ± 44 µmol/L, and 87 ± 16 µmol/L, respectively, whereas the mean ARG/ORN ratio was 3.58 ± 0.69. After adjusting for age, sex, and estimated glomerular filtration rate, there were no significant correlations between plasma HOMOARG and ARG or ORN concentrations, and between HOMOARG concentrations and the ARG/ORN ratio. Similarly, there were no significant correlations between plasma LYS and the ARG/ORN ratio (Table 5).

**Table 5:** Partial correlations, adjusted for age, sex, and estimated glomerular filtration rate, between plasma homoarginine, lysine, arginine, and ornithine concentrations, and the arginine/ornithine ratio (Arg/Orn)^*^

	Homoarginine	Lysine	Arginine	Ornithine	Arg/Orn
Homoarginine	—	r = 0.16 P = 0.31	r = 0.13 P = 0.38	r = −0.01 P = 0.92	r = 0.05 P = 0.73
Lysine	r = 0.16 P = 0.31	—	r = 0.30 P = 0.05	r = 0.26 P = 0.10	r = 0.02 P = 0.92
Arginine	r = 0.13 P = 0.38	r = 0.30 P = 0.05	—	r = 0.40 P = 0.007	r = 0.41 P = 0.006
Ornithine	r = −0.01 P = 0.92	r = 0.26 P = 0.10	r = 0.40 P = 0.007	—	r = −0.63 P < 0.001
Arg/Orn	r = 0.05 P = 0.73	r = 0.02 P = 0.92	r = 0.41 P = 0.006	r = −0.63 P < 0.001	—

^*^n = 46.

## Discussion

Here we investigated the inhibitory potential of HOMOARG on arginase enzyme activity using a newly developed, robust, sensitive, and highly selective UPLC-MS assay that measured ORN formation from recombinant human arginase 1 and arginase 2. Additionally, we investigated the hypothesis that HOMOARG serves as a substrate of these enzymes. The effects of HOMOARG on arginase activity were compared to those of other endogenous compounds serving either as negative (PRO, AG, ADMA, SDMA and L-NMMA) or positive (LYS) controls. Furthermore, we assessed the associations between plasma concentrations of HOMOARG and the ARG/ORN ratio in a cohort of healthy older adults without significant disease states and/or pharmacological treatment that may affect the outcomes of interest. In our study, HOMOARG showed significant inhibitory effects towards arginase 1 and 2 activity at concentrations that are considerably higher than those reported clinically in plasma (2.01 ± 0.67 µM) or in the cytoplasm (2.37 ± 2.28 µM) of peripheral blood mononuclear cells^23^. Furthermore, there were no significant associations between plasma HOMOARG concentrations and the ARG/ORN ration in the human study.

Several studies have recently reported an inverse correlation between HOMOARG plasma concentrations and the risk of cardiovascular events and overall mortality^1–8^. In some of these reports, arginase inhibition was proposed as a potential molecular mechanism accounting for the protective effect of HOMOARG in the cardiovascular system. NO is a key modulator of endothelial function and vascular homeostasis. By inhibiting arginase activity, HOMOARG would increase the availability of ARG as a substrate for NO synthase (NOS) and, therefore, NO availability^1,7^. While our *in vitro* data confirm the inhibitory potential of HOMOARG on both arginase 1 and arginase 2, the lack of significant inhibition at physiological concentrations questions the biological and clinical significance of arginase inhibition as a key mechanism accounting for the observed cardio-protective effects of HOMOARG. To determine if HOMOARG was acting as a substrate for arginase 1 or arginase 2 we investigated the formation of LYS as the product from the reaction, instead of ORN^24^. Increasing concentrations of HOMOARG did not result in increasing concentrations of LYS. Therefore, we propose HOMOARG is not a substrate of arginase 1 or arginase 2 under our experimental conditions.

Numerous studies have reported the kinetic characterisation of arginase 1 and arginase 2 activity^11–13,15,17,25,26^. By and large, published methods adopted colorimetric techniques for measuring urea formation in enzymatic reactions with ARG utilised as the substrate. Despite the advantages of this approach, such as low costs and high sensitivity, there are also significant limitations, in particular, poor specificity and reproducibility. By using mass spectrometry to detect and measure ORN as a product of arginase activity, our assay benefits from the inherent high selectivity and specificity of this technique. Another potential advantage of our approach is the use of un-purified protein in the form of EDTA-free lysates from HEK293T cells recombinantly expressing arginase 1 or arginase 2. This approach more closely mimics the *in vivo* environment by including the complexity of a cellular system. Furthermore, since our cellular lysates did not include chelating agents, and no purification steps were required to isolate the arginase 1 and arginase 2 proteins, manganese supplementation in kinetic experiments was not required. This allowed the use of phosphate buffer in the experimental system instead of “non-physiological” Tris-HCl. To demonstrate this point, we compared arginase activity in the presence and absence of manganese. Of particular note, we observed the rate of ORN formation was 20% higher in incubations undertaken in the absence of manganese, relative to samples in which 0.5 mM manganese chloride was supplemented to the incubation mixture. Despite the absence of manganese supplementation, the parameters derived from our experimental system were similar to those previously reported. For arginase 1 we determined the *K*_m_ to be 3.3 ± 0.2 mM, which is within the range of 1–9 mM previously reported for arginase 1^10–13,18,27^. Similarly, for arginase 2 the *K*_m_ value was 1.9 ± 0.1 mM, which is within the range of 1–7 mM previously reported^15,17,21,27^.

The methylated arginines ADMA, SDMA and L-NMMA, chemical analogues of the substrate ARG and important modulators of the nitric oxide pathway, were confirmed as poor arginase inhibitors in agreement with a previous study^18^. Similarly, no significant inhibition was observed with PRO and AG. LYS reduced arginase 1 and arginase 2 activity by 37–42% and 67–78% at concentrations of 1 and 10 mM, respectively, when the inhibition experiments were conducted at the *K*_m_ determined for ARG. A similar effect was observed at physiological ARG concentrations with 10 mM LYS inhibiting arginase 1 activity by 81% and arginase 2 activity by 88%. HOMOARG at concentrations of 1 and 10 mM was also effective in inhibiting arginase 1 and arginase 2 activity at concentrations of 0.1 and 2 mM (Table 3).

A full kinetic characterisation of LYS and HOMOARG as arginase 1 and arginase 2 inhibitors was performed and, consistent with the report of Ikemoto *et al*.^12^, the *K*_i_ values for arginase 1 inhibition by LYS and HOMOARG were found to be 1.8 and 6.1 mM, respectively (Ikemoto *et al*. reported *K*_i_ = 2.5–2.7 mM for LYS and *K*_i_ = 5.0–5.2 mM for HOMOARG). However, both LYS and HOMOARG exhibited a higher inhibitory potential on arginase 2 than previously reported, with IC_50_ and *K*_i_ values for LYS and HOMOARG of 0.9 and 0.5 mM (Colleluori *et al*. reported a *K*_i_ = 7 mM)^15^ and of 2.5 mM and 1.7 mM, respectively (Colleluori and Ash reported a *K*_i_ of 39 mM)^17^.

Similar to HOMOARG, none of the experiments performed showed inhibition of the arginase isoenzymes when LYS was used at concentrations close to the reported circulating values (276 ± 120 µM)^22^.

It is important to emphasise that the results of enzymatic studies in isolation cannot easily translate into the complexity of a living system and account for other related and/or competing enzymatic pathways^28,29^. However, when the experimental conditions are accurately chosen to match those present *in vivo* (particularly in terms of temperature, pH, buffer composition, and ionic strength), the findings can be used to speculate the physiological behaviour of the enzyme. Furthermore, good correlations are observed between *in vitro* kinetic experiments and *in vivo* K_cat_ using approaches that combine computational flux predictions and proteomics data^30^.

Using a cohort of healthy older adults, we further tested the hypothesis that plasma HOMOARG concentrations may be positively associated with the ARG/ORN ratio, a proposed indicator of arginase activity^31^. The lack of a significant correlation between HOMOARG concentrations and the ARG/ORN ratio in our study is in contrast to a previous study by Marz *et al*. that reported a significant positive correlation (r = 0.32; P =  < 0.001) in 3,305 subjects with high cardiovascular risk^1^. There are several significant differences between the two studies: 1) the ARG/ORN ratio in our study (3.58 ± 0.69) is significantly higher than the ratio reported by Marz *et al*. (1.47 ± 0.42); (2) The study by Marz *et al*. included patients with diabetes and hypertension. Interestingly, both these conditions are associated, *per se*, with an increase in arginase activity;^32,33^ and perhaps most importantly, (3) our cohort includes only healthy subjects, while the LURIC cohort comprised individuals with different pathological conditions, extremes of renal function and subjects undertaking different medications, all conditions that affect arginase activity^34^.

Although arginase inhibition is unlikely to be a key mechanism involved in the putative cardioprotective effects of HOMOARG, other mechanisms may play a role. For example HOMOARG is reported to be an alternative substrate for the enzyme NOS^20,35^, and despite its low affinity for the enzyme, it can directly increase NO availability^1^. Likewise, HOMOARG is a substrate of the different members of the cationic amino acid transporter (CAT) family of transporters and significantly inhibits the uptake of ARG by CAT-1, thus increasing ARG availability for NOS^36^. Furthermore, it may reduce blood pressure by facilitating the excretion of nitrate^1,37^.

In conclusion, HOMOARG-mediated arginase 1 and arginase 2 inhibition was observed at concentrations that are significantly higher than those observed in plasma or serum and in the cytoplasm. In support of this observation, there was no association between plasma HOMOARG and the ARG/ORN ratio in a cohort of healthy older adults. As such, arginase inhibition is unlikely to play a significant role in the protective effects of HOMOARG against cardiovascular risk and mortality.

## Methods

### *In vitro* studies

The experimental protocols for the assessment of arginase 1 and 2 inhibition *in vitro*, described below, were approved by the Institutional Biosafety Committee of Flinders University (IBC No 2009-08). All methods were performed in accordance with the relevant guidelines and regulations.

### Data availability

The datasets generated during and/or analysed during the current study are available from the corresponding author on reasonable request.

## Materials

Deuterated L-ornithine (ORN-d6), NG, N′G-dimethyl-L-arginine dihydrochloride (SDMA), and L-NG-monomethyl arginine (L-NMMA) acetate were obtained from Sapphire Bioscience (Sapphire Bioscience, Redfern, Australia). High purity water was obtained using a MilliQ Synergy UV Ultrapure water system (Merck Millipore, Sydney, Australia). Acetonitrile (LC-MS Grade), 2-propanol and formic acid (HPLC Grade) were obtained from Merck Millipore (Merck Millipore, Melbourne, Australia). All other laboratory grade chemicals and reagents were purchased from Sigma-Aldrich (Sigma-Aldrich, Sydney, Australia).

Stock solutions of 1 M ARG, 100 mM ORN, LYS, PRO, AG, HOMOARG and 10 mM L-NMMA, SDMA and ADMA, together with a 1 mg/mL stock solution of ORN-d6 were prepared in purified water. These solutions were stored frozen at −20 °C. The working internal standard solutions were prepared by diluting the 1 mg/mL ORN-d6 stock solution with water to 300 µM or 1200 µM final concentration. These working internal standard solutions were stored frozen at −20 °C.

### Arginase 1 and arginase 2 cloning and expression

The C-terminal cMYC-FLAG-tagged human arginase 1 (NM_000045) and arginase 2 (NM_001172) coding sequences (CDS) were purchased from Origene (OriGene Technologies, Rockville, MD) and shuttled into the pEF-IRES(6) mammalian expression vector. Cells were transfected with the pEF-IRES-arginase 1 or with the pEF-IRES-arginase 2 expression constructs (4 μg) using Lipofectamine2000 in OptiMEM (Invitrogen, CA, USA). The stable expression of arginase 1 and arginase 2 was achieved in HEK293T cells using puromycin as the selectable antibiotic.

A single batch of recombinant arginase 1 and arginase 2 was used in all experiments to avoid batch-related variability. Cultured cells were grown to 90% confluence, harvested, washed in phosphate-buffered saline solution and lysed by sonication in phosphate buffer (0.1 M, pH = 7.4) supplemented with an EDTA-free complete protease inhibitor cocktail (Roche Diagnostic GmbH, Mannheim, Germany) and 1 mM PMSF. Cell lysates were centrifuged for 10 minutes at 4 °C at 18000 x*g* to remove cellular debris and the supernatant fractions aliquoted to avoid repeated freeze thaw cycles and subsequently stored at −80 °C until use. Protein concentrations were determined by the method of Lowry^38^.

### Western blot analysis

Sodium dodecyl sulfate (SDS)–polyacrylamide gel electrophoresis was performed on cell lysates using 10% acrylamide gels to separate the denatured protein (120 V), and then transferred to Trans-Blot® Transfer Medium pure nitrocellulose (BIORAD; 0.45 μm; 100 V). Membranes were blocked in 4% (w/v) non-fat milk in Tris-buffered saline Tween20 for 90 mins with the primary immunodetection of arginase 1 and arginase 2 proteins achieved by probing blots with anti-arginase 1 (Santa Cruz, CA; 1:1000), anti-arginase 2 (Santa Cruz, CA; 1:1000,), and anti-FLAG (Sigma-Aldrich, Sydney, Australia; 1:5000) antibodies. Subsequently, blots were incubated with the corresponding peroxidase conjugated secondary antibodies (1:2000, 1 hour), with immunoreactivity detected using the SuperSignalWest Pico Chemiluminescent (ECL) HRP substrate (Thermo Fisher Scientific, Victoria, Australia) and imaged using the ImageQuant LAS-400 image reader (Fujifilm, Japan).

### Analytical instrumentation

ORN, LYS and ARG separation, detection and quantification were performed on a Waters ACQUITY^TM^ Ultra Performance LC™ system coupled to a Waters Premier quadrapole time of flight (qToF) mass spectrometer (Waters, Sydney, Australia). The electrospray ionisation (ESI) source was operated in positive ionisation mode (V+) and data collected over 10 min in ToF MS mode between 50 and 1000 Da with an instrument scan time of 1 sec and inter-scan delay of 0.1 sec. The mass spectrometer parameters are shown in Table 6. Instrument control, data acquisition and data processing were performed using Waters MassLynx software (version 4.1, Waters, Sydney, Australia).

**Table 6:** Mass spectrometer instrument settings.

Instrument Parameter	Setting
Capillary voltage (kV)	3.2
Sampling cone voltage (eV)	14.0
Extraction cone voltage (eV)	5.0
Source temperature (°C)	100
Desolvation temperature (°C)	300
Cone gas flow(L/Hr)	50.0
Desolvation gas flow (L/Hr)	400.0
Collision energy	3.0
Collision Cell Entrance	2.0
Collision Exit	−10.0
Collision Gas Flow (mL/min)	0.6

### UPLC-MS analysis of ORN

ORN was separated from the cellular lysate components on a Waters Atlantis^TM^ HILIC column (2.1 × 150 mm, 3 µm, Waters, Sydney, Australia) held at 35 °C. The mobile phase comprised acetonitrile containing 0.1% v/v formic acid (mobile phase A) and 0.1% formic acid in a solution of 10% v/v acetonitrile in water (mobile phase B) at a flow rate of 0.4 mL/min. Initial conditions were 70% mobile phase A, 30% mobile phase B. The proportion of mobile phase B was increased linearly to 45% over 7.4 min, held at 45% for 1 min then returned to 30% for 1.6 min to re-establish equilibrium prior to injection of the following sample.

The method was assessed for linearity, reproducibility and sensitivity (limit of detection and limit of quantitation). Limit of quantitation was determined as the concentration of product giving 10:1 signal to noise ratio, while the limit of detection was assigned as the concentration corresponding to 3:1 signal to noise ratio. Calibration standards comprised of ORN concentrations ranging from 0 to 200 µM spiked into the incubation mixture and were extracted and reconstituted following the same procedure used for incubation samples.

### Arginase activity assay

ORN formation was determined at 37 °C in a total incubation volume of 0.1 mL using 12 × 75 mm borosilicate glass tubes. Incubation mixtures contained HEK293T cell lysate expressing recombinant human arginase 1 (0.05 mg/mL) or arginase 2 (0.1 mg/mL), phosphate buffer (0.05 M, pH 7.4) and ARG (0 to 15 mM).

Following a 5-min pre-equilibration period reactions were initiated by the addition of the cell lysate solution in 0.05 M phosphate buffer to a pre-equilibrated aqueous solution of the substrate (ARG). Following incubation at 37 °C the reaction was terminated by the addition of 300 μL 0.1% formic acid in 2-propanol and 10 µL of the assay internal standard ORN-d6 was added to each reaction tube. For the arginase 1 assay the incubation time was 10 min and the assay working internal standard solution was 1200 μM ORN-d6. For arginase 2 assays the incubation time was 20 min and the ORN-d6 working internal standard concentration was 300 μM

The samples were vortex mixed (20 sec) and cooled in an ice/water bath for 10 min prior to centrifugation (5 min, 18,000 × g, room temperature) to precipitate the proteins. An aliquot of the supernatant layer was diluted with a 7:3 mobile phase A/mobile phase B solution into glass UPLC vial inserts. For UPLC-MS analysis the arginase 1 sample supernatant layer was diluted 1:20 with the mobile phase solution and 2 μL of each diluted sample was injected for ORN analysis whereas arginase 2 analysis was performed by diluting the supernatant layer 1:10 and injecting 5 μL for analysis.

To determine if manganese supplementation was necessary, we performed an experiment using arginase 1 (0.1 mg/mL), phosphate buffer (0.05 M, pH 7.4), ARG (0 to 10 mM) and manganese chloride (0 or 0.5 mM). Incubation samples were incubated for 20 min and protein was precipitated and samples prepared for ORN analysis as previously described.

Samples were maintained at 15 °C in the auto-sampler prior to analysis.

### Arginase inhibition

Incubation mixtures comprised of HEK293T cell lysate expressing arginase 1 (0.05 mg/mL) or arginase 2 (0.1 mg/mL), phosphate buffer (0.05 M, pH 7.4), inhibitor (0, 0.1, 1, 10, 100, 1000 or 10000 μM) and ARG (0.1, 2 or 3 mM). Following a 5-min pre-incubation of the potential inhibitor with arginase 1 or arginase 2, reactions were initiated by the addition of a pre-equilibrated solution of the substrate ARG. Protein precipitation and preparation for ORN analysis was performed as described above (see arginase activity assay).

### IC_50_ experiments

For arginase 1 assay incubation mixtures comprised of arginase 1 cell lysate (0.05 mg/mL), phosphate buffer (0.05 M, pH 7.4), LYS or HOMOARG (0–10 mM) and ARG (3 mM). Incubation, protein precipitation and preparation for ORN analysis were performed as previously described (see arginase inhibition and arginase activity assay).

For arginase 2 assay incubation mixtures comprised of arginase 2 cell lysate (0.1 mg/mL), phosphate buffer (0.05 M, pH 7.4), LYS (0–2.5 mM) or HOMOARG (0–5 mM) and ARG (2 mM). Incubation, protein precipitation and preparation for ORN analysis were performed as described above (see arginase inhibition and arginase activity assay).

### *K*_i_ experiments

For arginase 1 incubation mixtures comprised of arginase 1 cell lysate (0.05 mg/mL), phosphate buffer (0.05 M, pH 7.4), LYS or HOMOARG (0–10 mM) and ARG (0.6–15 mM). Incubation, protein precipitation and preparation for ORN analysis were performed as previously described (see arginase inhibition and arginase activity assay).

For arginase 2 incubation mixtures comprised of arginase 2 lysate (0.1 mg/mL), phosphate buffer (0.05 M, pH 7.4), LYS (0–2 mM) or HOMOARG (0–4 mM) and ARG (0.5–8 mM). Incubation, protein precipitation and preparation for ORN analysis was performed as described above (see arginase inhibition and arginase activity assay). Sample were diluted 3:17 in mobile phase solution prior injection.

## *In vivo* studies

### Homoarginine, lysine and arginine/ornithine ratio in humans

Plasma concentrations of HOMOARG, LYS, ARG, and ORN were measured, using an Aquity UPLC (Waters, Sydney, Australia) coupled to a qToF Premier high-resolution mass spectrometer (Waters, Sydney, Australia), in 50 healthy community dwelling adults > 65 years (27 males and 23 females) participating in a study investigating the effects of fish oil consumption on adaptations to resistance exercise^39^. Study participants had no previous history of significant disease and were not on regular medications, barring one female participant treated with angiotensin converting enzyme inhibitors for hypertension and one male participant treated with allopurinol for gout. The study was approved by the University of Aberdeen College of Life Sciences and Medicine Ethics Review Board (CERB/2011/6/644) and registered at clinicaltrials.gov (identifier NCT02843009). Each participant provided written informed consent prior to the study.

### Data analysis

Kinetic constants (*K*_m_, V_max_) for ORN formation were derived from model fitting the Michaelis-Menten equation to experimental data using the nonlinear curve fitting software EnzFitter (version 2.0.18.0: Biosoft, Cambridge, UK). Kinetic data are the mean of three singlicate experiments.

IC_50_ values for arginase 1 and arginase 2 inhibition by LYS and HOMOARG were determined by fitting the IC_50_ equation to experimental data using the same software and data are the mean of two singlicate experiments.

Fitting of the experimental data to noncompetitive, mixed and uncompetitive inhibition models was performed using EnzFitter and comparison of the statistical values was used to determine the best fit. *K*_i_ values were derived from fitting the competitive inhibition equation to experimental data. K_i_ data are the mean of three singlicate experiments.

Goodness of fit of all equations was assessed from the F statistic, 95% confidence intervals, r^2^ value, and standard error of the parameter fit.

Associations between HOMOARG, ARG, and ORN concentrations, and ARG/ORN ratios in the human study were assessed by partial correlations, adjusted for age, sex, and estimated glomerular filtration rate. Analyses were performed using IBM SPSS Statistics Version 23, Release 23.0.0.2 (SPSS Inc., Armonk, NY, USA). A two-sided P < 0.05 indicated statistical significance.

## Electronic supplementary material

Supplementary Information
