# Supplementary material for: Homoarginine and inhibition of human arginase activity: kinetic characterization and biological relevance
Source: Sci Rep. 2018 Feb 27;8:3697. doi: 10.1038/s41598-018-22099-x (PMC5829263; doi:10.1038/s41598-018-22099-x)
Supplement: Supplementary file 1 — Supplementary Information [file 41598_2018_22099_MOESM1_ESM.pdf]

Homoarginine and inhibition of human arginase activity: kinetic characterization and biological relevance

S. Tommasi[1], D.J. Elliot[1], M. Da Boit[2], S.R. Gray[3], B.C. Lewis[1,4], A.A. Mangoni[1*]

[1]Department of Clinical Pharmacology, College of Medicine and Public Health, Flinders University and Flinders Medical Centre, Adelaide, Australia;

[2]Faculty of Health and Life Sciences, De Montfort University, Leicester, United Kingdom;

[3]Institute of Cardiovascular and Medical Sciences, University of Glasgow, Glasgow, United Kingdom;

[4]Flinders Centre for Innovation in Cancer, College of Medicine and Public Health, Flinders University, Adelaide, Australia;

SUPPLEMENTARY FIGURES

anti-arginase 1

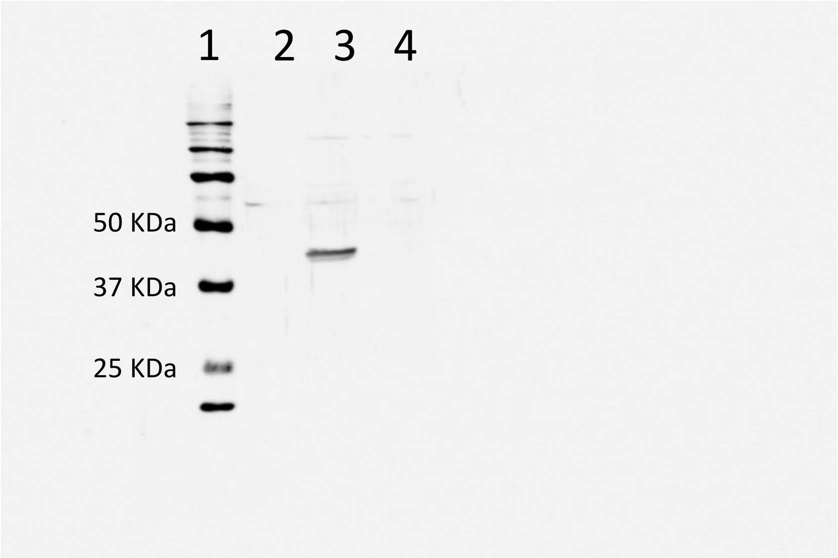

Figure S1. Expression of recombinant arginase 1 and arginase 2 in HEK293T cell lysate as shown by representative western blots performed using anti-arginase 1. Each blot shows: molecular markers (lane 1), untransfected HEK293T cell lysate (lane 2, 100 µg), arginase 1 lysate (lane 3, 30 µg), and arginase 2 lysate (lane 4, 30 µg).

anti-arginase 2

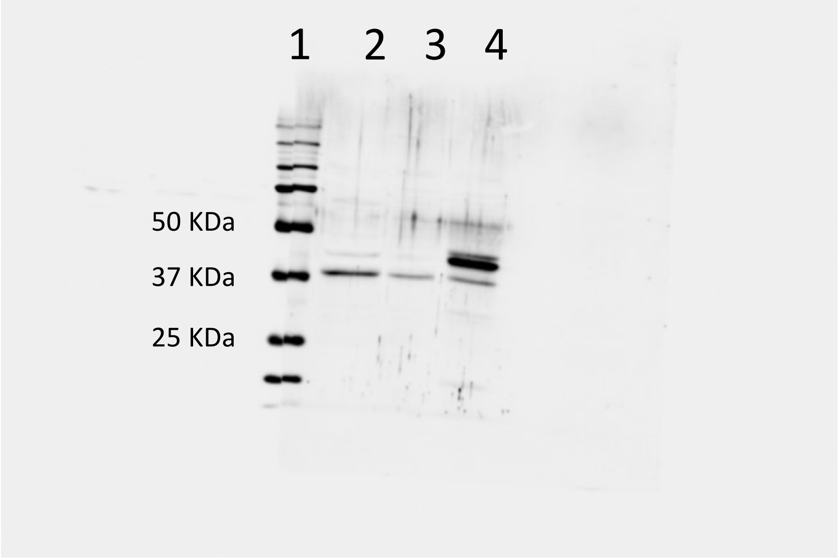

Figure S2. Expression of recombinant arginase 1 and arginase 2 in HEK293T cell lysate as shown by representative western blots performed using anti-arginase 2. Each blot shows: molecular markers (lane 1), untransfected HEK293T cell lysate (lane 2, 100 μg), arginase 1 lysate (lane 3, 30 μg), and arginase 2 lysate (lane 4, 30 μg).

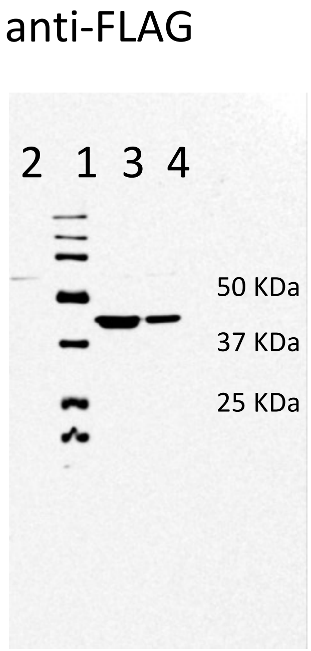

Figure S3. Expression of recombinant arginase 1 and arginase 2 in HEK293T cell lysate as shown by representative western blots performed using anti-FLAG. Each blot shows: molecular markers (lane 1), untransfected HEK293T cell lysate (lane 2, 100 μg), arginase 1 lysate (lane 3, 30 μg), and arginase 2 lysate (lane 4, 30 μg).
